# Curcumin reduces prostaglandin E2, matrix metalloproteinase-3 and proteoglycan release in the secretome of interleukin 1β-treated articular cartilage

**DOI:** 10.12688/f1000research.2-147.v2

**Published:** 2013-08-20

**Authors:** Abigail L Clutterbuck, David Allaway, Pat Harris, Ali Mobasheri

**Affiliations:** 1 School of Veterinary Medicine and Science, The University of Nottingham, Sutton Bonington Campus, LE12 5RD, UK; 2 WALTHAM Centre for Pet Nutrition, Waltham-on-the-Wolds, Melton Mowbray, LE14 4RT, UK; 3 Medical Research Council-Arthritis Research UK Centre for Musculoskeletal Ageing Research, The University of Nottingham, Nottingham, NG7 2UH, UK; 4 Arthritis Research UK Pain Centre, The University of Nottingham, Nottingham, NG7 2UH, UK; 5 Arthritis Research UK Centre for Sport, Exercise, and Osteoarthritis, The University of Nottingham, Nottingham, NG7 2UH, UK; 6 Faculty of Medicine and Health Sciences, The University of Nottingham, Sutton Bonington Campus, LE12 5RD, UK; 7 School of Pharmacy and Life Sciences, University of Bradford, Bradford, BD7 1DP, UK; 8 Center of Excellence in Genomic Medicine Research, King Abdulaziz University, Jeddah, 21589, Saudi Arabia; 9 The D-BOARD European Consortium for Biomarker Discovery, The University of Nottingham, Nottingham, NG7 2UH, UK

## Abstract

**Objective:** **Curcumin (diferuloylmethane) is a phytochemical with potent anti-inflammatory and anti-oxidant properties, and has therapeutic potential for the treatment of a range of inflammatory diseases, including osteoarthritis (OA). The aim of this study was to determine whether non-toxic concentrations of curcumin can reduce interleukin-1beta (IL-1β)-stimulated inflammation and catabolism in an explant model of cartilage inflammation.

**Methods:** **Articular cartilage explants and primary chondrocytes were obtained from equine metacarpophalangeal joints. Curcumin was added to monolayer cultured primary chondrocytes and cartilage explants in concentrations ranging from 3μM-100μM. Prostaglandin E _2_ (PGE _2_) and matrix metalloproteinase (MMP)-3 release into the secretome of IL-1β-stimulated explants was measured using a competitive ELISA and western blotting respectively. Proteoglycan (PG) release in the secretome was measured using the 1,9-dimethylmethylene blue (DMMB) assay. Cytotoxicity was assessed with a live/dead assay in monolayer cultures after 24 hours, 48 hours and five days, and in explants after five days.

**Results:** **Curcumin induced chondrocyte death in primary cultures (50μM p<0.001 and 100μM *p<*0.001) after 24 hours. After 48 hours and five days, curcumin (≥25μM) significantly increased cell death ( *p<*0.001 both time points). In explants, curcumin toxicity was not observed at concentrations up to and including 25μM after five days. Curcumin (≥3μM) significantly reduced IL-1β-stimulated PG ( *p*<0.05) and PGE _2_ release ( *p<*0.001) from explants, whilst curcumin (≥12μM) significantly reduced MMP-3 release ( *p<*0.01).

**Conclusion: **Non-cytotoxic concentrations of curcumin exert anti-catabolic and anti-inflammatory effects in cartilage explants.

## Introduction

Osteoarthritis (OA) involves destruction of articular cartilage by a combination of mechanical injury, inflammatory mediators and proteolytic enzyme activity ^1^. The high cost and potential negative side effects of conventional pharmacotherapy, i.e. non-steroidal anti-inflammatory drugs (NSAIDs), has stimulated interest in natural plant products with anti-inflammatory properties, as an alternative or adjunct to conventional therapy ^2^. These products are being investigated for potential efficacy in a wide range of disorders with an inflammatory component, including arthritis and cancer ^3, 4^.

Curcumin (diferuloylmethane) is a polyphenol found in turmeric derived from the rhizomes of *Curcuma longa*. Curcumin is traditionally known for its powerful anti-inflammatory and anti-oxidant properties. At concentrations between 50 and 100μM it has been shown to have anti-inflammatory properties via its suppressive effects on I kappa B kinase (IKK) activity and consequently the nuclear factor-kappa B (NF-κB) signaling pathway in various cell types, including chondrocytes ^5, 6^. However, published work suggests that curcumin is cytotoxic to both primary chondrocytes ^7^ and transformed chondrocyte cell lines ^8^ at 50μM and above. Primary cells in their initial passages can be more phenotypically and genotypically relevant than transformed cells and are more applicable to the clinical setting ^9^. Therefore, the cytotoxicity observed in chondrocyte cell lines may be a consequence of the transformation induced by the SV-40 virus. The hypothesis to be tested in this study was that curcumin exerts anti-inflammatory and anti-catabolic effects on interleukin-1beta (IL-1β)-stimulated cartilage explants at non-cytotoxic concentrations. Accordingly, we evaluated the concentrations at which a commercially available curcumin formulation (sourced from Sigma-Aldrich) was cytotoxic to primary equine chondrocytes in both monolayer and explant cultures and determined whether non-toxic concentrations could reduce proteoglycan (PG) loss and inflammatory mediator production in an *in vitro* model of early OA.

## Materials and methods

### Tissues

Macroscopically normal articular cartilage samples were obtained from weight-bearing regions of the metacarpophalangeal joints of eleven horses of mixed breed, age and sex. The joint tissues were sourced from UK-based abattoirs. The joint tissues were sourced from UK-based abattoirs and veterinary practices. Animals were euthanized for non-research purposes either in accordance with Welfare of Animals (Slaughter or Killing) Regulations 1995 or the Veterinary Surgeons Act with owner consent. Approval for the use of clinical materials was obtained from the local Ethical Review Committee. Full depth cartilage from six animals was taken for explant culture and thin cartilage shavings were used for chondrocyte isolation from the remaining five animals. Cartilage samples from each animal were kept separate throughout. Cartilage shavings were aseptically harvested into low glucose Dulbecco’s modified Eagle’s medium (DMEM; HyClone, Thermo Fisher Scientific, Loughborough, UK) containing 4% penicillin/streptomycin (Sigma-Aldrich, Gillingham, UK) before washing in phosphate-buffered saline (PBS) containing 10% penicillin/streptomycin (Sigma-Aldrich, Gillingham, UK) for 20 minutes.

### Chondrocyte isolation and culture

Thin cartilage slices were digested overnight in 0.1% collagenase type I (Sigma-Aldrich, Gillingham, UK) at 37°C and 5% CO _2_. The resulting cell suspension was filtered and washed before undergoing first expansion in low glucose DMEM with 2% penicillin/streptomycin and 10% fetal bovine serum (FBS). Once confluency was reached, cells were passaged into 12-well plates. Only first and second passage confluent cells were used in this study.

### Experimental design–monolayer cultures

Culture media was removed and replaced with treatment media (1ml/well). Control wells contained media alone (low glucose DMEM with 2% penicillin/streptomycin and 10% FBS), which formed the base for the other treatments. The NSAID carprofen (100μg/ml; Rimadyl ^®^, Pfizer, Sandwich, UK) was included as a positive control, due to its anti-inflammatory effects on chondrocytes ^10^. The nitric oxide donor, sodium nitroprusside (SNP; Sigma-Aldrich, Gillingham, UK) dissolved in DMEM (50mM) was used as a positive control for inducing cell death ^11^. Stock solutions of curcumin (100mM; C1386, Sigma-Aldrich, Gillingham, UK) were prepared in cell culture grade dimethyl sulfoxide (DMSO; Sigma-Aldrich, Gillingham, UK) and diluted in DMEM to 1mM. From this 1mM stock, experimental concentrations of curcumin (3μM, 6μM, 12μM, 25μM, 50μM and 100μM) were prepared in DMEM and added to the appropriate wells. A DMSO control containing a volume equivalent to that found in the highest curcumin concentration was included on each plate to ensure that any observed effects were not due to the carrier solvent. Curcumin was not used to pretreat the cells and explants prior to the addition of IL-1β. Curcumin and IL-1β were added simultaneously to the cultures. The plates were incubated at 37°C and 5% CO _2_. Cytotoxicity was assessed after 24 hours, 48 hours and five days.

### Cytotoxicity assays–monolayer chondrocytes

Chondrocyte viability was assessed using a commercially available live/dead assay (Invitrogen, Paisley, Scotland, UK) that utilizes calcein AM and ethidium homodimer-1 to identify live and dead cells, respectively. Media was removed and centrifuged (Eppendorf, Eppendorf rotor) at 10,000 rpm for 25 seconds at room temperature. The resulting pellet of detached cells (if any) was washed and resuspended in 20μl PBS. Adherent cells in the wells were washed in PBS before adding 20μl of the detached cell suspension to the appropriate wells and incubating in calcein AM (2μM) and ethidium homodimer-1 (4μM) in PBS (Sigma-Aldrich, Gillingham, UK) for 30 minutes at room temperature. Fluorescence was detected and captured using an inverted contrasting microscope (Leica DM IL, Leica Microsystems Ltd, Wetzlar, Germany) with Leica Application Suite imaging software (Version 2.4.0 R1, Leica Microsystems Ltd). Six random fields of view of live and dead cells were taken per well (magnification×100). Live and dead cells were counted with ImageJ Software (National Institutes of Health, Bethesda, MD) and the percentage of dead cells (expressed as a percentage of the total number of cells present) was calculated at 24 hours, 48 hours and five days for each treatment.

### Cartilage explant culture

Full depth cartilage shavings were cut into 3mm discs. Three discs per well from the same animal were placed in 24-well plates containing 1ml of culture medium (serum-free low glucose DMEM supplemented with 2% penicillin/streptomycin) and allowed to equilibrate overnight at 37°C under 5% CO _2_. The following day, culture media was replaced with fresh media before the experiment began.

### Experimental design–cartilage explants

Cartilage from three animals was used for curcumin viability studies, and cartilage from three different animals was used for the remaining studies. All plates contained 1ml culture media, which formed the base for other treatments and acted as a control for each plate. Explant viability studies were performed by adding curcumin (12μM, 25μM and 100μM) prepared in DMEM as described above to the appropriate wells (one well per animal per treatment). SNP (50mM) was used as a positive control for cell death. The remaining 3 wells per animal per treatment were used to determine PG release from unstimulated explants in response to curcumin.

Experiments aimed at assessing the anti-catabolic and anti-inflammatory effects of curcumin were conducted by incubating explants in culture media containing recombinant equine IL-1β (R&D Systems, Abingdon, UK) (10ng/ml) and various concentrations of curcumin (3μM, 6μM, 12μM, 25μM and 50μM) prepared in IL-1β-treated media. Carprofen (100μg/ml) was prepared in IL-1β-treated media and included as a positive control. DMSO controls were performed previously and found to have no effect on PG release from both IL-1β-stimulated and unstimulated cartilage explants at volumes equivalent to that found in the highest curcumin concentration (data not shown). Plates were incubated at 37°C and 5% CO _2_ for five days. After five days, explants were immediately assayed for cytotoxicity or frozen at -20°C with their corresponding supernatants for subsequent secretome assays.

### Cytotoxicity assays–explant cultures

After five days, chondrocyte viability was assessed using the live/dead assay. Explants were washed in PBS then incubated with calcein AM (2μM) and ethidium homodimer-1 (8μM) in PBS for 30 minutes at room temperature. A confocal microscope (Leica TC SP2) was used to detect and measure fluorescence in 15μm z-sections though each explant (magnification×10).

### DMMB assays

For evaluation of matrix PG release we used the metachromatic dye 1,9 dimethylmethylene blue (DMMB) to quantify the amount of sulfated glycosaminoglycans (GAGs) into the medium. Cartilage discs were digested in papain (Sigma-Aldrich, Gillingham, UK) for 16 hours. Papain-digested cartilage and their corresponding supernatants were assayed in 96-well plates using the DMMB (Sigma-Aldrich, Gillingham, UK) method as previously described ^12^. Shark chondroitin sulfate (Sigma-Aldrich, Gillingham, UK) was used as a standard (0–70μg). DMMB solution (200µl) was added to samples and standards (40μl). The plate was read (Multiskan Ascent, Thermo Labsystems, Basingstoke, UK) using Ascent Software (version 2.6, Thermo Labsystems, Basingstoke, UK). Total PG release was obtained from a spectrophotometric reading of the digested cartilage and its corresponding supernatant at 540nm. Percentage of PG release from the total PG content of the explants was calculated by dividing the supernatant value from the total PG release for each well.

### Prostaglandin E _2_ (PGE _2_) immunoassay

A competitive immunoassay kit (R&D Systems, Abingdon, UK) was used to measure PGE _2_ release according to the manufacturer’s instructions. Standards (19.6–1250pg/ml), supernatant samples and reagents were added to a 96-well plate coated in goat anti-mouse polyclonal antibody and incubated for 19 hours at 6°C. The plate was washed and developed with 200μl substrate solution per well in the dark at room temperature for 20 minutes. A stop solution was added and the plate was read immediately (Multiskan Ascent, Thermo Labsystems, Basingstoke, UK) at 450nm with wavelength correction set at 540nm using Ascent Software (version 2.6, Thermo Labsystems, Basingstoke, UK).

### Western blot analysis of MMP-3 release

The protein content of explant supernatants from the PG and PGE _2_ assays was quantified and aliquots containing 50µg protein were freeze-dried overnight. The resultant pellets were resuspended in 37µl sample buffer (NuPAGE Lithium dodecyl sulfate sample buffer (4×) and electrophoresed on precast 4–12% Bis-Tris 10-well gels (Invitrogen, Paisley, Scotland, UK) under denaturing and reducing conditions. Proteins were transferred to 0.45µm polyvinylidene fluoride (PVDF) membranes (GE Healthcare, Little Chalfont, UK) and blocked with 5% (w/v) non-fat milk with Tris-Buffered saline (TBS) containing 0.1% (v/v) Tween20 for 1 hour. Membranes were incubated with a goat polyclonal antibody to matrix metalloproteinase 3 (stromelysin) (MMP-3; Abcam, Cambridge, UK) diluted 1:1,000 in 5% (w/v) non-fat milk at 4°C overnight. After washing, membranes were incubated for two hours at room temperature with a secondary anti-goat antibody (1:10,000; Dako, Cambridge, UK). Membranes were washed and chemiluminescence detected using ECL+ on a Typhoon Trio+ Variable Mode Imager (both GE Healthcare, Little Chalfont, UK). Densitometric quantification of MMP-3 bands was performed using ImageJ software. Relative band intensity in comparison to controls was measured for samples from each animal.

### Statistical analysis

Data were statistically analyzed using a one-way analysis of variance (ANOVA) with Tukey’s *post hoc* test (GraphPad InStat, version 3.05, GraphPad Software Inc., La Jolla, CA). Statistical significance was set at *p*<0.05. Graphs were plotted with GraphPad Prism (version 4, GraphPad Software Inc).

For chondrocyte viability quantification, results are expressed as the mean number of dead cells per field of view per treatment ± standard error of the mean (SEM). PG release percentage and PGE _2_ values are reported as means of combined animals ± SEM. For MMP-3 quantification analysis, relative intensity values were reported as means of 3 combined animals ± SEM.

## Results

### Curcumin is cytotoxic to primary chondrocytes at 25μm and above

**Figure 1.**
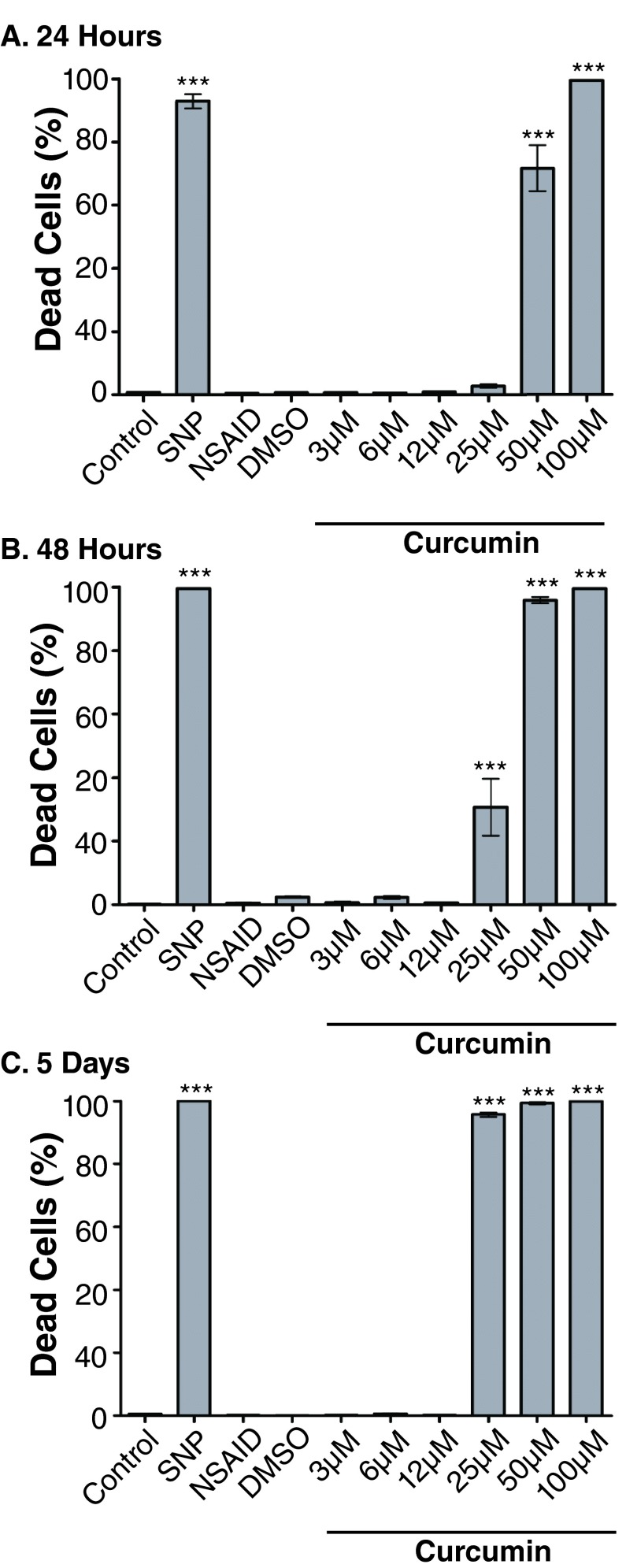
Curcumin significantly increases chondrocyte death after 24 hours ( **A**) at 50μM and 100μM compared to control indicated by *** ( *p*<0.001). After 48 hours ( **B**) and five days ( **C**), curcumin (25μM) significantly increases chondrocyte death compared to controls. The nitric oxide donor, sodium nitroprusside (SNP) (50mM) also significantly increases chondrocyte death at 24 hours, 48 hours, and five days ( *p*<0.001) compared to control. DMSO at concentrations found in the 100μM curcumin treatment has no effect on chondrocyte death. Results are expressed as the mean number of dead cells per field of view per treatment ± SEM. Data presented are from 5 different animals with 2 technical replicates per animal.

Untreated controls retained a mean cell death percentage of less than 1% at 24 hours, 48 hours and five days ( Figure 1). DMSO controls and the NSAID, carprofen, did not significantly increase cell death compared to controls at all time points. SNP effectively induced cell death ( *p*<0.001, all time points) with mean cell death of 92.95 ± 2.29% at 24 hours, 99.6 ± 0.17% at 48 hours and 100 ± 0.00% at five days. Curcumin significantly increased cell death compared to controls after 24 hours at 50μM (71.75 ± 7.25%, *p*<0.001) and 100μM (99.55 ± 0.12%, *p*<0.001). After 48 hours a significant increase in toxicity compared to controls was seen at 25μM (30.67 ± 8.94%, *p*<0.001), 50μM (95.9 ± 0.96%, *p*<0.001) and 100μM (99.59 ± 0.18%, *p*<0.001). After five days, curcumin (25μM) caused significant increases in cell death (95.71 ± 0.72%, *p*<0.001), 50μM (99.4 ± 0.39%, *p*<0.001) and 100μM (100 ± 0.00%, *p*<0.001).

### Curcumin is not cytotoxic to cartilage explant chondrocytes at 25μm after five days

**Figure 2.**
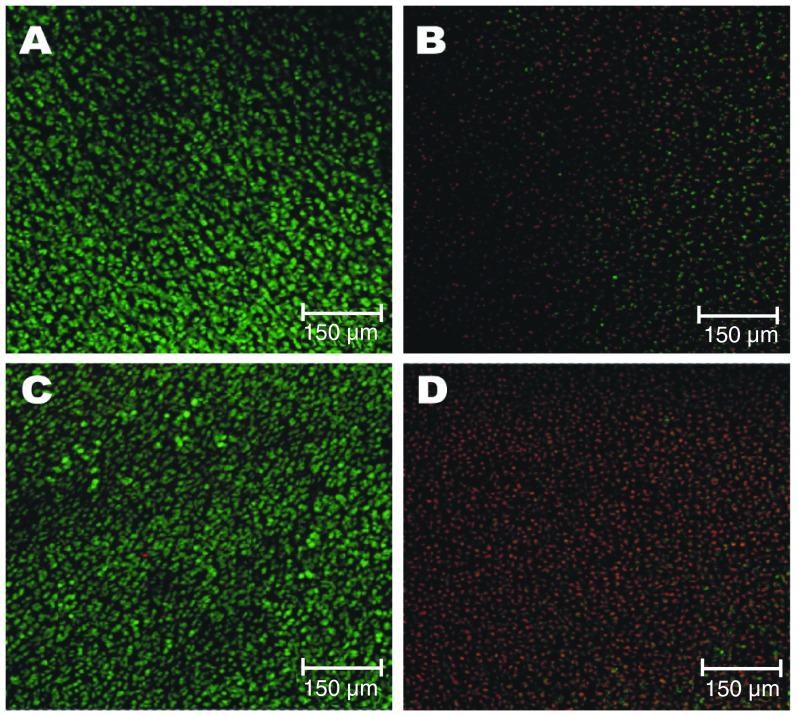
Three-dimensional confocal reconstructions of z stacks through cartilage explants (15μm sections) after five days in culture. Control ( **A**) consists of culture media with 2% penicillin/streptomycin. Sodium nitroprusside (SNP) (50mM) ( **B**) is a positive control for cell death. Treatments consist of curcumin in culture media at 25μM ( **C**) and 100μM ( **D**). Images are representative of explants in the treatments wells for each animal. Green staining indicates live metabolizing cells and red staining highlights the nuclei of dead cells. Data are from 3 different animals, with one technical replicate per animal.

After five days in culture without serum supplementation, explants retained fully viable chondrocytes as indicated by the abundant green staining and lack of red staining in the controls ( Figure 2). SNP induced cell death in the explants as shown by the increased red staining and fewer, less vibrant green stained cells in comparison to the controls. Curcumin (25μM)-treated explants retained large numbers of green stained cells, showing no detriment to the viability of chondrocytes within cartilage explants after five days in culture. However, the large amount of red nuclei staining to the chondrocytes in the 100μM curcumin-treated explants indicates that curcumin was highly cytotoxic at this concentration.

### Curcumin does not influence PG release from unstimulated cartilage explants

**Figure 3.**
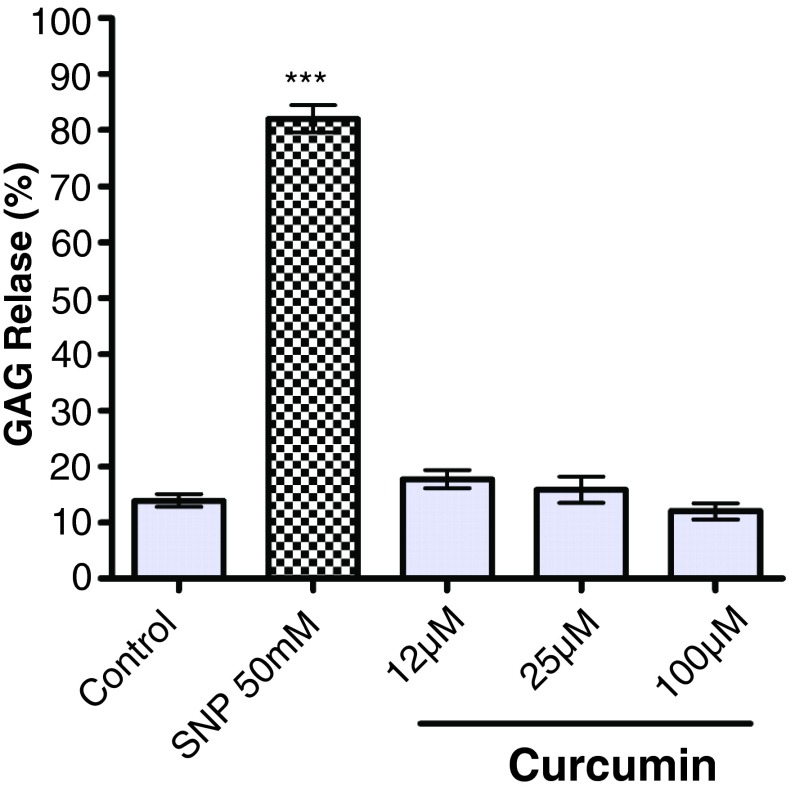
Effect of curcumin on proteoglycan (PG) release from unstimulated cartilage explants. Control column indicates cartilage discs incubated in the culture medium alone. Values are reported as the mean of three animals per treatment ± SEM. Data are from 3 different animals, with three technical replicates per animal. Significance compared to control is indicated by *** ( *p*<0.001). Sodium nitroprusside (SNP) (50mM) significantly increases PG release compared to control ( *p*<0.001). Curcumin does not alter PG release from the explants compared to control at concentrations of 12µM, 25µM or 50µM. PG loss is expressed as sulfated glycosaminoglycan (GAG) release.

Control explants released 13.91 ± 1.13% of the total PG content of the cartilage into the media over five days (range: 159–414μg PG/ml of culture supernatant) ( Figure 3). SNP (50mM) significantly increased matrix PG release to 82.01 ± 2.43% ( *p*<0.001) after the same period. Curcumin did not significantly alter PG release from the explants after five days at 12μM, 25μM and 100μM.

### Curcumin significantly suppresses IL-1β-stimulated PG and PGE _2_ release in cartilage explants

**Figure 4.**
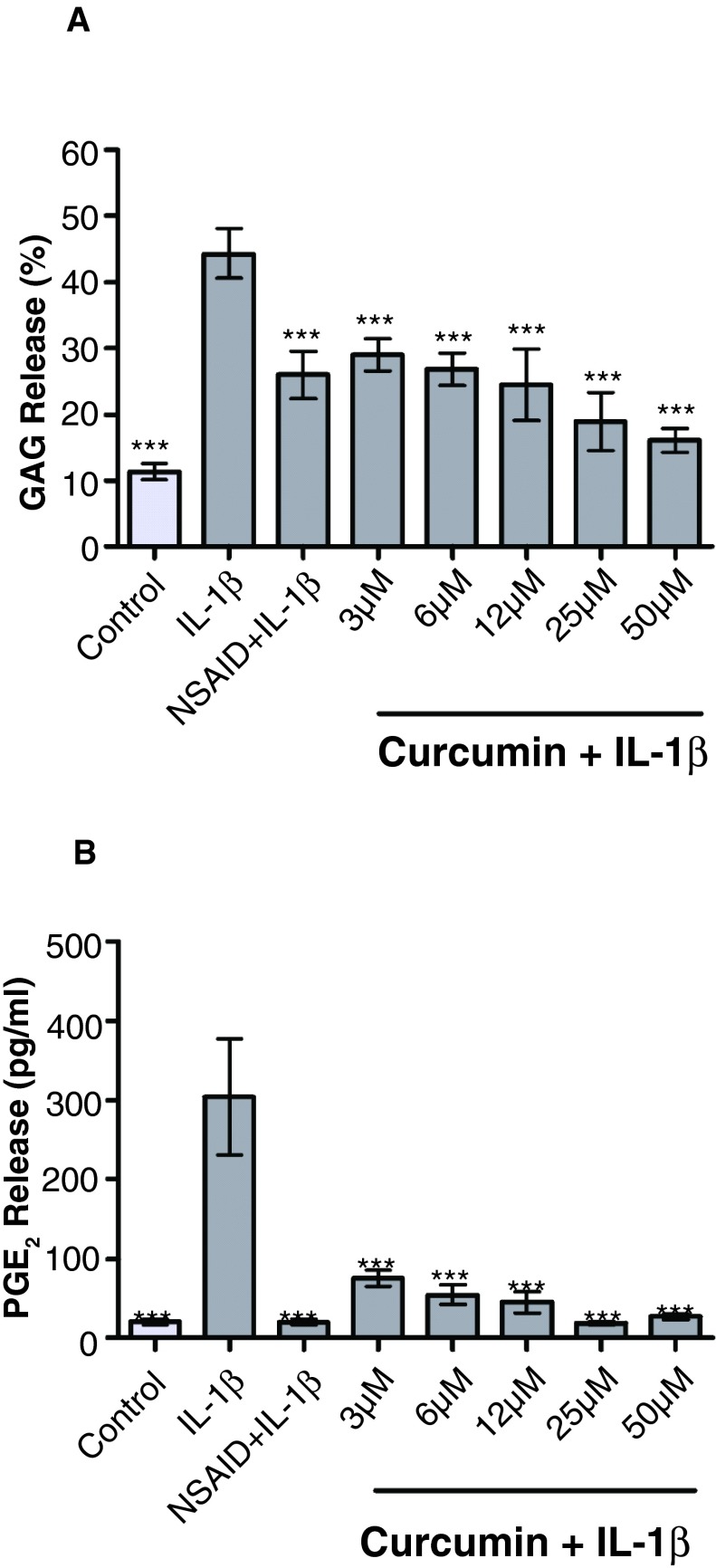
Effect of curcumin on PG release ( **A**) and prostaglandin E2 (PGE _2_) ( **B**) from interleukin (IL)-1β-stimulated cartilage explants (dark grey columns) (n=3). Control (light grey column) indicates cartilage discs incubated in the culture medium alone. Values are reported as the mean of three animals per treatment ± SEM. Data are from 3 different animals, with three technical replicates per animal. Significance compared to IL-1β is indicated by * ( *p*<0.05), ** ( *p*<0.01) and *** ( *p*<0.001). IL-1β significantly increases proteoglycan (PG) ( *p*<0.001) and PGE _2_ ( *p*<0.001) release compared to control. The non-steroidal anti-inflammatory drug (NSAID) carprofen (100µg/ml) significantly reduces IL-1β-stimulated PG ( *p*<0.01) and PGE _2_ ( *p*<0.001) release. Curcumin significantly reduces IL-1β-stimulated PG release at 3µM ( *p*<0.05), 6µM ( *p*<0.01), 12µM ( *p*<0.01), 25µM ( *p*<0.001) and 50µM ( *p*<0.001). IL-1β-stimulated PGE _2_ release is also significantly reduced at all curcumin concentrations ( *p*<0.001). PG loss is expressed as sulfated glycosaminoglycan (GAG) release.

Control explants released 11.38 ± 1.26% of the total PG content of the cartilage into the media over five days (range: 86–323μg PG/ml of culture supernatant) ( Figure 4A). IL-1β (10ng/ml) significantly increased PG release to 44.31 ± 3.75% of total PG content compared to control ( *p*<0.001). The NSAID carprofen (100μg/ml) significantly reduced IL-1β-stimulated PG release to 25.96 ± 3.59% of total PG content ( *p*<0.01). Curcumin significantly decreased IL-1β-stimulated PG release in the explants to 28.98 ± 2.45% at 3µM ( *p*<0.05), 26.84 ± 2.49% at 6µM ( *p*<0.01), 24.51 ± 5.42% at 12µM ( *p*<0.01), 18.91 ± 4.36% at 25µM ( *p*<0.001) and 16.05 ± 1.82% at 50µM ( *p*<0.001). PGE _2_ release into the media of unstimulated explants was 20.35 ± 3.67pg/ml ( Figure 4B). These levels were significantly increased ( *p*<0.001) to 303.3 ± 73.38pg/ml by the addition of recombinant IL-1β (10ng/ml). The NSAID significantly attenuated this effect, reducing levels to 19.72 ± 3.74pg/ml ( *p*<0.001). Curcumin also significantly reduced IL-1β-stimulated PGE _2_ release to 75.48 ± 10.68pg/ml at 3μM ( *p*<0.001), 54.72 ± 12.41pg/ml at 6μM ( *p*<0.001), 45.65 ± 13.31pg/ml at 12μM ( *p*<0.001), 18.36 ± 2.38pg/ml at 25μM ( *p*<0.001) and 26.73 ± 3.52pg/ml at 50μM ( *p*<0.001).

### Curcumin exerts dose-dependent effects on IL-1β-stimulated MMP-3 release in cartilage explants

**Figure 5.**
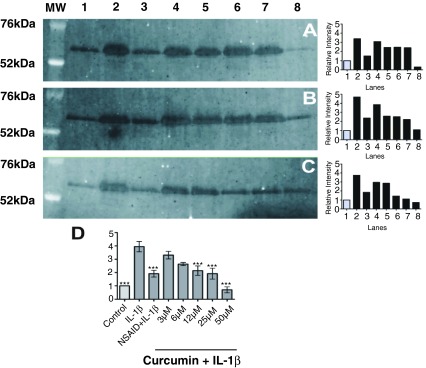
Western blot images and quantifying graphs of matrix metalloproteinase (MMP)-3 in the cartilage explant supernatants from three animals ( **A**, **B**, and **C**). The lane on the left of the images shows the molecular weights (MW) of standard markers (Invitrogen, Paisley, Scotland, UK) in kilodaltons (kDa). Graph **D** shows the relative intensity of bands from all three animals with labeled treatment axes. Values are reported as the mean of three animals per treatment ± SEM. Significance compared to interleukin (IL)-1β is indicated by ** ( *p*<0.01) and *** ( *p*<0.001). All lanes contain equal volumes of protein (50µg protein per lane). Unstimulated cartilage explants release low levels of MMP-3 as shown by the controls in lane 1. Secretion of MMP-3 from explants is significantly increased ( *p*<0.001) by IL-1β (10ng/ml) (Lane 2). The non-steroidal anti-inflammatory drug (NSAID) carprofen (100μg/ml) (Lane 3) significantly reduces IL-1β-stimulated MMP-3 secretion ( *p*<0.01) to near control levels. Curcumin at concentrations of 3μM (Lane 4) and 6μM (Lane 5) does not significantly reduce IL-1β-stimulated MMP-3 release from the explants. However, a significant reduction is observed at 12μM ( *p*<0.01) (Lane 6), 25μM ( *p*<0.01) (Lane 7) and 50μM ( *p*<0.001) (Lane 8) from explants in a dose dependent manner. The extent of reduction differs between animals (one technical replicate was used from each animal).

Control explants released low levels of MMP-3, which were significantly increased by the addition of IL-1β ( *p*<0.001) ( Figure 5). The addition of the NSAID carprofen significantly reduced IL-1β-stimulated MMP-3 levels ( *p*<0.01) in all animals to near that of controls. Curcumin showed a dose-dependent significant effect on reducing the IL-1β-stimulated release at 12μM ( *p*<0.01), 25μM ( *p*<0.01) and 50μM ( *p*<0.001). However, the concentration at which this reduction became apparent differed between animals. For example, animal C, showed a clear reduction in MMP-3 secretion at curcumin concentrations of 12μM and above, whereas in animals A and B, the equivalent reduction was not seen until curcumin concentrations of 50μM were used. The most apparent reduction in MMP-3 secretion was always seen at 50μM with levels near to that of the controls in all animals.

**Effect of curcumin on prostaglandin E2, matrix metalloproteinase-3 and proteoglycan release in articular cartilage**

*11 Data Files*

http://dx.doi.org/10.6084/m9.figshare.724793

## Discussion

Recent research has produced conflicting results regarding the efficacy and cytotoxicity of curcumin *in vitro* ^13– 15^. This study attempted to address the issue of curcumin cytotoxicity and its anti-inflammatory effects in equine cartilage explants and monolayer chondrocyte cultures. The results of this investigation suggest that curcumin induces chondrocyte death at concentrations of 25μM and above in monolayer chondrocytes after 48 hours and five days.

The cytotoxic effects of curcumin (50μM) have been observed and documented in other cell types, notably tumor cell lines ^16^. Induction of apoptosis through cytochrome *c* release and subsequent caspase activation is thought to be a key chemopreventive effect of curcumin in cancer studies ^17^. In agreement with this, curcumin (50μM) has also been shown to reduce the viability of an immortalized human chondrocyte cell line after 24 hours ^8^. The results from this study suggest that this is also the case with primary equine chondrocytes. However, a recent study on primary human chondrocytes found that curcumin (50μM) did not reduce cell viability and successfully inhibited IL-1β-induced cytotoxicity as demonstrated by a 3-(4,5-dimethylthiazol-2-yl)-2,5-diphenyltetrazolium bromide (MTT) assay ^18^. This could be due to species-specific differences in chondrocyte susceptibility to curcumin, or equally likely, the different sources of curcumin used. This highlights the importance of assessing cytotoxicity alongside anti-inflammatory assays, with the same formulation of curcumin in each individual study.

In contrast to the monolayer cell cultures, curcumin (25μM) did not induce cytotoxicity in chondrocytes within cartilage explants after five days, although toxicity was seen at 100μM. Within cartilage, chondrocytes are embedded in a complex extracellular matrix (ECM) across which diverse diffusion gradients and fluid flow occurs ^19^. This could account for the differing toxicity threshold between monolayer cultures and explants. Chondrocytes are more vulnerable to cell death when they are no longer encased within a matrix, as cell adhesion and integrin attachments are important factors in promoting cell survival ^20^. Thus, without a protective ECM, monolayer chondrocytes may be more exposed and susceptible to external agents in the culture media than those within explants, as has been shown with bupivacaine ^21^. Therefore, cytotoxicity data from monolayer cultures may not reflect cytotoxic changes in 3-dimensional culture models and explant cultures.

For the purposes of addressing cytotoxicity in this study, a live/dead stain was selected to detect chondrocyte death as it can quickly provide qualitative and quantitative data on both monolayer and 3-dimensional chondrocyte cultures. The method does not distinguish between apoptosis and necrosis as the mode of cell death and although this information was not required for this study, it could be a useful addition to future studies. It should be noted here that cell death in monolayer cells can be underestimated, as dead cells can detach and be removed with the media. However, this risk was mitigated by centrifuging the discarded media so that the resulting pellet of detached cells could be resuspended and returned to the well before the live/dead stain reagents were added.

Once the uppermost observed safe level of curcumin was determined, the effect of curcumin on cartilage catabolism and inflammation was examined in explants. No significant effect of curcumin alone on matrix PG release from normal cartilage explants at both safe and toxic concentrations was observed. This suggests that curcumin does not alter the basal level of PG released from unstimulated cartilage explants in culture, even at cytotoxic concentrations. Interestingly, in these unstimulated explants, the two treatments that caused cell death had different effects on PG release: SNP (50mM) caused extensive PG loss into the media whereas curcumin (100μM) did not alter PG release compared to controls. PG loss does not directly lead to cell death in cartilage explants ^22^, thus the PG loss caused by SNP is unlikely to be primarily a consequence of cell death. SNP generates nitric oxide (NO), ceramide and cyanide, which aside from causing apoptosis, reduces proteoglycan and collagen synthesis by chondrocytes and increases inflammatory mediator production and MMP activity ^23– 25^ resulting in cartilage matrix loss. Conversely, although curcumin (100μM) induces cell death, it is likely to involve a mechanism that does not induce the release of any proteases. Curcumin is known to reduce the release of inflammatory mediators, MMPs and NO ^6, 26^. We have shown in this study that cytotoxic levels of curcumin reduce IL-β-stimulated PGE _2_ and MMP-3 release. The reduced production of inflammatory mediators and catabolic enzymes suggests that it is unlikely to induce extensive PG loss as demonstrated in both IL-1β-treated and untreated cartilage explants in this study.

Curcumin at 3μM and above significantly reduced PG loss from IL-1β-stimulated explants. This reduction in PG loss was seen at concentrations that were non-detrimental to chondrocyte viability in explants (i.e. 25μM and under) as well as cytotoxic concentrations (100μM). Previously, we observed that curcumin (100μM) significantly reduced human IL-1β-stimulated PG release from cartilage explants, but this effect was not seen at 10μM ^27^. The differences in concentration efficacy between these two studies may be linked to individual variation or the source of cytokines used. Our previous study used human IL-1β on equine explants, whereas the current study used equine recombinant IL-1β. Despite a fairly conserved sequence homology between species, the use of species-specific IL-1β is more representative of the *in vivo* situation and thus may explain the difference in results seen between studies.

The involvement of pro-inflammatory cytokines and MMPs in OA is well documented ^28, 29^. MMP-3, also known as stromelysin, is produced by chondrocytes in OA cartilage tissue ^30^. MMP-3 gene expression is up-regulated in response to IL-1β stimulation in chondrocytes ^31^. Similarly, MMP-3 protein expression is increased by the addition of IL-1β to cartilage explants ^32^. MMP-3 was chosen as a marker of cartilage degradation in this study, as there is convincing evidence for a role for MMP-3 in cartilage destruction in OA. MMP-3 is induced by IL-1β in joint inflammation, and has the capacity to degrade collagens, proteoglycans and activate other procollagenases ^33^. MMP-3 and two other MMPs including MMP-1 and MMP-13 can also cleave the aggregating proteoglycan aggrecan at the interglobular domain ^34– 36^. Curcumin reduced MMP-3 secretion at concentrations as low as 12μM in some animals, and as high as 50μM in others, suggesting some animal-to-animal variability. However, when treated with 50μM curcumin, IL-1β-stimulated explants from all the animals used secreted MMP-3 levels that were lower than, or equivalent to, unstimulated control explants. This is in agreement with a previous study showing that curcumin (50μM) effectively reduced MMP-3 levels in IL-1β-stimulated-human chondrocyte lysates ^37^. However, lower curcumin concentrations have been reported as effective in cartilage from human OA patients post mortem, where curcumin reduced MMP-3 release into the media of IL-1β-stimulated chondrocytes at 15μM and in cartilage explants at 5μM ^38^. Many variables may account for the difference in effective curcumin concentrations, including individual variation, pre-existing joint pathology and the explant model used. It should be noted that many MMPs, including collagenases, matrilysin (MMP-7), and other stromelysins (e.g. MMP-10), are involved in osteoarthritic cartilage degradation ^39– 41^. Thus, the reduction in MMP-3 secretion would contribute to, but not totally account for, the reduction in PG release from IL-1β-stimulated cartilage explants.

The reported anti-inflammatory effects of curcumin have stimulated increasing interest in its potential for the treatment of inflammatory disorders ^42^. Curcumin is thought to exert its anti-inflammatory effects through reducing COX-1 ^43^, COX-2 ^44^, and microsomal PGES expression ^16^, thus preventing PGE _2_ release. This is most likely due to its inhibitory effect on the upstream NF-κB-signaling pathway, which promotes PGE _2_ production via upregulating the COX and PGES genes ^45^. In this study, curcumin at concentrations of 3μM and over significantly reduced PGE _2_ release in response to equine IL-1β (10ng/ml). This anti-inflammatory effect is consistent with previous work in other cell culture models, such as rat peritoneal macrophages where curcumin (10µM) inhibited PGE _2_ release by 45% ^46^, and in BV2 microglial cells where curcumin (10µM and 20µM) significantly reduced PGE _2_ release in response to lipopolysaccharide (LPS) (0.5µg/ml) ^47^. The reduction in PGE _2_ levels in response to IL-1β in our study may be attributed to the inhibitory effects of curcumin on the NF-κB pathway. Curcumin (50µM) has been shown to inhibit various steps of the NF-κB pathway, such as IL-1β-dependent phosphorylation of p65; nuclear-translocation of p65; and IκBα phosphorylation in IL-1β-stimulated human chondrocytes ^6^. Thus by inhibiting NF-κB, curcumin prevents the downstream inflammatory effects of COX-2 expression and PGE _2_ synthesis.

Although curcumin was able to effectively attenuate the catabolic effects of IL-1β in this model, many other biochemical and biomechanical factors are capable of inducing cartilage inflammation and degeneration; these include compressive stress caused by overloading or traumatic injury of the joint ^48, 49^ and different cytokines such as tumor necrosis factor alpha (TNF-α) alone and in combination with oncostatin M (OSM) ^50^. Further studies are required to determine whether curcumin can reduce inflammation and degeneration generated by combinations of different catabolic stimuli.

The anti-inflammatory and anti-catabolic effects of curcumin shown in this study support the existing evidence that curcumin may be supportive to joint health ^13– 15^. However, the bioavailability of curcumin is thought to be relatively low ^51^ and efforts to determine whether chemically modified versions of curcumin may improve bioavailability merit further investigation ^13, 52^.

In summary, although this study found that curcumin at concentrations of 25μM and above is cytotoxic to monolayer chondrocytes after five days in culture, lower concentrations effectively antagonize PG and PGE _2_ release *in vitro* and exert a potent anti-inflammatory effect on cartilage explants treated with IL-1β. Achieving micromolar concentrations of curcumin in the synovial joint, or to the pericellular matrix of chondrocytes embedded deep within the avascular articular cartilage is highly unlikely. Controversial issues associated with the metabolism and bioavailability of curcumin highlight the need for caution when extrapolating *in vitro* data for translational research and clinical trials. Nevertheless, our results to date suggest that a commercially available curcumin formulation at 12μM and below, has no obvious cytotoxic effects on primary chondrocytes after five days in culture. More importantly, concentrations as low as 3μM were able to effectively reduce IL-1β-stimulated cartilage degradation and inflammatory mediator production. This study supports existing evidence to suggest that curcumin may be a suitable adjunct to conventional drugs for the treatment of inflammatory and degenerative disorders such as OA. If curcumin is to be used as an anti-inflammatory supplement, further research is required to establish its bioavailability and physiologically relevant serum and synovial concentrations *in vivo* in humans and animals.
