# Supplementary material for: Curcumin reduces prostaglandin E2, matrix metalloproteinase-3 and proteoglycan release in the secretome of interleukin 1β-treated articular cartilage
Source: F1000Res. 2013 Aug 20;2:147. Originally published 2013 Jul 4. [Version 2] doi: 10.12688/f1000research.2-147.v2 (PMC3901452; doi:10.12688/f1000research.2-147.v2)
Supplement: Peer review history

# Peer review: Referee response for version 2

**Published:** 2013-09-09

**Authors:** Stefan Toegel

**Affiliations:** 1 Medical University of Vienna, Vienna, Austria

The authors have adequately addressed my remarks on this submission and I do not have any further concerns regarding the acceptance of the article .

I have read this submission. I believe that I have an appropriate level of expertise to confirm that it is of an acceptable scientific standard.

# Response

**Published:** 2013-09-09

**Authors:** Ali Mobasheri

**Affiliations:** School of Veterinary Medicine and Science, Jointly appointed at the University of Nottingham and the University of Bradford, UK

The authors wish to thank Dr. Toegel for the constructive comments that have helped us improve the manuscript and for approving the second version of the paper.

# Peer review: Referee response for version 2

**Published:** 2013-09-06

**Authors:** Oliver Grundmann

**Affiliations:** 1 University of Florida, Gainesville, FL, USA

The revised article submitted by Clutterbuck *et al*. has addressed my concerns and I do not have any further comments.

I have read this submission. I believe that I have an appropriate level of expertise to confirm that it is of an acceptable scientific standard.

# Response

**Published:** 2013-09-09

**Authors:** Ali Mobasheri

**Affiliations:** School of Veterinary Medicine and Science, Jointly appointed at the University of Nottingham and the University of Bradford, UK

The authors wish to thank Dr. Grundmann for his insightful comments and for approving the second version of the paper.

# Peer review: Referee response for version 1

**Published:** 2013-07-15

**Authors:** Stefan Toegel

**Affiliations:** 1 Medical University of Vienna, Vienna, Austria

In the present report, Clutterbuck and co-workers describe the cytotoxic and anti-inflammatory effects of curcumin in equine cartilage explants and chondrocytes. Their study demonstrates that curcumin is a potent inhibitor of IL1ß-induced PGE2, MMP3 and GAG release from cartilage at non-toxic concentrations. I found this article very interesting to read as it highlights important aspects of practical relevance for research on curcumin activity in cartilage. I do have a few remarks that might help to somewhat improve the manuscript.

**Suggested revisions**

1. The Figure legends should include detailed information on the number of technical and biological replicates used. 
2. The Methods should clarify whether curcumin and IL1ß were added simultaneously to the cells/explants or whether curcumin was used to “pretreat” the cells/explants prior to the addition of IL1ß.
3. In the results section the authors state: “The most apparent reduction in MMP-3 secretion was always seen at 50μM with levels near to that of the controls in all animals.” This issue is also discussed in the 'discussion section later on. However, as Figure 1 demonstrates, significant cytotoxic results are expected from applying this concentration. The results should therefore be discussed in light of this finding.
4. A reference protein (loading control) should be included in the Western Blot images in Figure 5.
5. Did the authors check the Gaussian distribution of the data before applying ANOVA statistics?

I have read this submission. I believe that I have an appropriate level of expertise to confirm that it is of an acceptable scientific standard.

# Response

**Published:** 2013-08-11

**Authors:** Ali Mobasheri

**Affiliations:** School of Veterinary Medicine and Science, Jointly appointed at the University of Nottingham and the University of Bradford, UK

**Response to Dr. Stefan Toegel**

1. In response to Dr. Stefan Toegel’s review, we have included detailed information on the number of technical and biological replicates used in the revised figure legends.
  Fig 1 - 5 biological replicates (animals) and 2 replicates per animal 
  Fig 2 - 3 biological replicates (animals) and 1 replicate per animal
  Fig 3 - 3 biological replicates (animals) as already stated in the legend and 3 replicates per animal
  Fig 4 - 3 biological replicates (animals) as already stated in the legend and 3 replicates per animal
  Fig 5 - 3 biological replicates (animals) as already stated in the legend and 1 replicate per animal 
2. Curcumin was not used to “pretreat” the cells/explants prior to the addition of IL-1β. Curcumin and IL-1β were added simultaneously. The revised Materials and methods section now clarifies the fact that curcumin and IL-1β were added simultaneously to the cells and explants. 
3. This is a very good point. However, we are mainly talking about curcumin at 25uM and under being non cytotoxic, yet effective. 
4. We did not include a reference protein in the western blots, as there are no proteins that can effectively be used as reference proteins in the secretome. However, we did perform a protein assay to ensure that equal quantities of protein were loaded on the polyacrylamide gels before western blotting.
5. No, we did not check the Gaussian distribution of the data before applying ANOVA statistics.

# Peer review: Referee response for version 1

**Published:** 2013-07-09

**Authors:** Oliver Grundmann

**Affiliations:** 1 University of Florida, Gainesville, FL, USA

This interesting article submitted by Clutterbuck *et al.* is a well written and designed research paper. I do have a few comments that would help to clarify the research for readers.

 

**Suggested revisions:**

 

1. In the methods section the authors should clearly state how many repetitions have been used for each experiment and each condition – this is not clear at times. Presumably at least 3 repetitions per assay per condition were made in order to derive the statistics but this should be specified. 
2. In figures 3 and 4A, the % of proteoglycan release is mentioned and graphed – how is this related to the total PG content if the variability appears to be rather high from the 80s to over 300 μg/mL? This needs to be clarified. 
3. In figure 5, did the authors use a reference protein to ensure that the same total protein content was used for each band? If so, this should be included as a reference in Figure 5.
4. In the discussion section, the authors mention that curcumin in different preparations can present with varying effects. Since curcumin is a pure substance isolated from Curcuma longa, it will have the same chemical properties and therefore – given that it is properly dissolved like in this study using DMSO in non-toxic concentrations – then it should not be different from any other study. If the authors refer to a Curcuma longa extract then it would be a different issue worth discussing.
5. Furthermore, and of great importance, the authors briefly mention the low bioavailability of curcumin *in vivo* in humans – how does that compare to the concentrations that were used in these experiments? What would be a good comparison blood concentration one could expect in humans after oral application of curcumin? This should be included in the discussion section to provide the reader with some perspective.

I have read this submission. I believe that I have an appropriate level of expertise to confirm that it is of an acceptable scientific standard.

# Response

**Published:** 2013-08-11

**Authors:** Ali Mobasheri

**Affiliations:** School of Veterinary Medicine and Science, Jointly appointed at the University of Nottingham and the University of Bradford, UK

**Response to Dr. Oliver Grundmann**  

1. In response to Dr. Grundmann’s comments we have added some additional points to clarify the research for the readers. In response to both reviewers we have included the number of technical and biological replicates used. At least 3 biological replicates were used in each experiment and some assays included multiple technical replicates. 
2. Regarding the proteoglycan release data, it can be argued that such results should be expressed as micrograms of glycosaminoglycan (GAG) per milligram of cartilage to account for variation in explant weight, as previously shown as either wet weight or dry weight. However, percentage GAG release has been used in previous studies and was selected as a more accurate measure. Expressing the results as a percentage means that the proportion of GAG released was quantified in relation to the total amount of GAG in each explant. Obviously, this is not the case when discussing the PGE _2_ results but although the data are expressed as a total value of PGE _2_ per ml, pilot studies showed that the explant weight variation was low, with a mean wet weight (± SEM) of 4.24 ± 0.10mg. Thus, the explants were of a similar weight and size. 
3. As highlighted in our response to Dr. Toegel, we did not include a reference protein in the western blots, as there are no reliable secreted proteins that can effectively be used as a loading control in secretome samples. Instead, we performed a protein assay to ensure that protein was equally loaded on the gels before western blotting. 
4. We agree with Dr. Grundmann’s assertion that pure curcumin isolated from *Curcuma longa* should have identical effects when dissolved in DMSO in cell culture studies as it should retain the same chemical properties. The curcumin used in this study is a widely used formulation from Sigma-Aldrich with a reported purity of approximately 70%. It could be argued that only pure curcumin should have been tested to ensure that the effects seen in the models were from curcumin alone. However, the curcumin used in this manuscript, has been used by several investigators on other cell types, and so there was a benefit in using it to enable comparisons with the published literature. It should be noted here that many commercially available curcumin preparations, including the preparation used in this manuscript, are often a mixture of three curcuminoids; curcumin (also known as diferuloylmethane), demethoxycurcumin and bisdemethoxycurcumin. Therefore, the term ‘curcuminoid’ may be more appropriate to describe curcumin preparations of this nature. However, for comparative purposes with published studies that use the term ‘curcumin’, and in the absence of analytical data determining the activity of various fractions of the curcuminoid constituents in the curcumin used in this study, the term curcumin was used to describe the commercially available formulation. We hope that Dr. Grundmann will be satisfied with this explanation. 
5. Finally, we wish to refer Dr. Grundmann to three recent review articles in which the corresponding author has addressed the issue of low curcumin bioavailability *in vivo* in human subjects (please refer to citations 13, 14 and 15 in this paper). These reviews have been cited in this manuscript. The range of concentrations used in these experiments was indeed higher than those that can be achieved *in vivo*. However, the pharmacokinetic profile of curcumin is characterized by its low serum concentration and limited tissue distribution. The available clinical data supports the notion that the serum concentration of curcumin peaks 1-2 hours after an oral dose in human subjects with peak serum concentrations of 0.5, 0.6 and 1.8 micromolar at doses of 4, 6 and 8 g/day. These are the most reliable serum concentrations that one might expect in humans after oral consumption of curcumin. Strategies to improve bioavailability include the use of sustained released drug delivery systems, liposome delivery systems and nanoparticles. The possibility is the intraarticular injection of biologically active doses of curcumin to metabolising chondrocytes and synoviocytes. However, there are no studies that have attempted intra-articular injection of high doses of curcumin.
